# The Relationships Between Multidimensional Schizotypy and Metacognitive Beliefs

**DOI:** 10.1177/00332941251314707

**Published:** 2025-01-18

**Authors:** Anchal Garg, Bruce D. Watt, Mary Rihan, Ahmed A. Moustafa

**Affiliations:** School of Psychology, Faculty of Society and Design, 3555 Bond University, Gold Coast, QLD, Australia; School of Social Sciences, 622667 Western Sydney University, Parramatta, NSW, Australia; School of Psychology, Faculty of Society and Design, Bond University, Gold Coast, QLD, Australia; Department of Human Anatomy and Physiology, Faculty of Health Sciences, University of Johannesburg, South Africa

**Keywords:** metacognition, at-risk mental states, psychosis, negative schizotypy, disorganised schizotypy, psychopathology

## Abstract

There has been a recent surge in schizotypy and metacognition research. Metacognition is an umbrella term for higher-order thought processes. Here, we focussed on maladaptive metacognitive beliefs, which are beliefs related to one’s thought processes and often play an important role in the preponderance of psychological disorders. Despite the extensive literature, relatively less is known about metacognitive beliefs in the context of multidimensional schizotypy, which consists of positive, negative, and disorganised dimensions and represents the milder forms of schizophrenia symptoms and vulnerability to developing schizophrenia. This is the first study that examined the links not only with positive schizotypy, but also with negative and disorganised schizotypy. We also attempted to circumvent the limitations related to schizotypy assessment in the previous studies by using a newly developed measure, the Multidimensional Schizotypy Scale (MSS), which was based on the current operationalisations of the construct. We examined the links in a non-clinical sample, including first-year psychology students and members of the wider community of an Australian university (*N* = 68). Self-report data was collected by administering the Metacognitions Questionnaire-30, MSS, and Marlowe-Crowne Social Desirability Scale to control the self-report measures’ social desirability biases. Results revealed positive correlations of maladaptive metacognitive beliefs not only with positive schizotypy, but also with negative and disorganised schizotypy. Our findings support the importance of examining schizotypy as a multidimensional construct. Further, our findings have implications for research and practice, including treatment modalities focusing on the modification of metacognitive beliefs for the prevention of schizophrenia and related conditions.

## Introduction

Metacognition is an umbrella term for higher-order thought processes (Livingston, 2003) and is commonly referred to as *thinking about one’s thinking* in the literature (Flavell, 1979). This construct has a wide appeal and applicability, which has resulted in research activities across several disciplines, such as psychology, education, and neurosciences (e.g., Norman et al., 2019). This far-reaching nature of metacognition has resulted in numerous operationalisations, and therefore, in this paper, we will focus on one subtype of metacognition: metacognitive beliefs.

### Metacognitive Beliefs

Metacognitive beliefs can be defined as the beliefs that are related to the interpretation, selection, and execution of specific thought processes (Cartwright-Hatton & Wells, 1997; Larøi & Van der Linden, 2005; Morrison, 2001). Such beliefs can be adaptive or maladaptive. Here, we focus on maladaptive beliefs, such as the beliefs about thought processes (e.g., “My memory can mislead me at times”), and the beliefs about the content of one’s thoughts (e.g., “It is bad to think certain thoughts”; Morrison, 2001; Wells & Cartwright-Hatton, 2004). These metacognitive beliefs are suggested to play an important role in psychological disturbances if they support irrational interpretations of thoughts, or unachievable goal states, or even biases in the allotment of mental resources (Ostefjells et al., 2015; Wells & Cartwright-Hatton, 2004).

We operationalise metacognitive beliefs based on the Self-Regulatory Executive Function Model (S-REF Model; Wells & Matthews, 1996). The S-REF model posits that a core Cognitive Attentional Syndrome (CAS) is linked to the maintenance of unhelpful thinking patterns that maintain clinical symptomatology and distress (Sellers et al., 2016). The CAS comprises of rumination, worry, threat monitoring, and engagement with unhelpful coping strategies, and arises from maladaptive metacognitive beliefs, which can be positive and negative in content (Wells & Cartwright-Hatton, 2004). Specifically, the unhelpful/maladaptive metacognitive beliefs comprise: (a) *positive beliefs about worry*, which focus on the usefulness of the CAS (e.g., “Worrying helps me to cope”), (b) *negative beliefs about the uncontrollability and danger of thoughts* (e.g., “My worrying is dangerous for me”), (c) *cognitive confidence* refers to beliefs about lack of confidence in one’s memory and attentional capacities (e.g., “I have a poor memory”), (d) *negative beliefs about the need to control thoughts* (e.g., “If I could not control my thoughts, I would not be able to function”), and (e) *cognitive self-consciousness* refers to beliefs about the predisposition to focus on one’s thought processes (e.g., “I constantly examine my thoughts”; Wells & Cartwright-Hatton, 2004). The five metacognitive beliefs are commonly examined using the *Metacognitions Questionnaire* (MCQ-65; Cartwright-Hatton & Wells, 1997), or the shortened version, the *Metacognitions Questionnaire-30* (MCQ-30; Wells & Cartwright-Hatton, 2004).

The metacognitive beliefs are transdiagnostic as they are prevalent across several clinical conditions, including major depressive disorder, generalised anxiety disorder, obsessive-compulsive disorder, eating disorders, and schizophrenia (e.g., Sun et al., 2017). Schizophrenia/psychosis is of considerable interest as these patients have been found to score higher on the MCQ-65/MCQ-30 compared to healthy controls (e.g., Sellers et al., 2017). Similarly, schizotypy is of significant interest as “schizotypy is thought to represent the phenotypic manifestation of the underlying vulnerability for schizophrenia-spectrum psychopathology that is expressed across a broad range from subclinical expression to the prodrome to schizophrenia-spectrum personality disorders to full-blown psychosis” (Kwapil et al., 2018, p. 209). Further, schizotypy is a valuable construct for understanding the aetiological and treatment modalities related to schizophrenia and related conditions (Kwapil & Barrantes-Vidal, 2015). Among non-clinical samples, metacognitive beliefs are associated with schizotypy traits, such as proneness to hallucinations and delusions (e.g., Stainsby & Lovell, 2014) and the total schizotypy score (Chan et al., 2015). Consequently, examining the links between metacognitive beliefs and schizotypy traits may provide unique insights into the internal mentation of individuals with schizophrenia, without confounds, such as hospitalisations or medications, and therefore, is the main focus of this paper (Kwapil & Barrantes-Vidal, 2015; Nelson et al., 2013).

### Multidimensionality of Schizotypy

Schizotypy is a multidimensional construct consisting of positive, negative, and disorganised dimensions (Kwapil & Barrantes-Vidal, 2015; Kwapil et al., 2018). Positive schizotypy refers to excessive or distorted functioning of normal processes, including disruptions in thought content, perceptual disturbances, and suspiciousness/paranoia (Kwapil et al., 2018). Negative schizotypy refers to diminution or deficit in one’s normal behaviour, such as alogia, anergia, and social disinterest (Kwapil et al., 2018). Disorganised schizotypy refers to disturbances in the organisation and expression of thoughts, speech, and behaviour, which could range from mild disturbances to formal thought disorders and extremely disorganised behaviours (Kwapil et al., 2018). Although there is not a widespread consensus in the literature regarding the number and nature of schizotypy dimensions, the three schizotypy dimensions mentioned above have been replicated across cultures (e.g., Fonseca-Pedrero et al., 2018).

Several measures have been developed to assess schizotypy, such as the Schizotypal Personality Questionnaire (SPQ; Raine, 1991) and the Oxford-Liverpool Inventory of Feelings and Experiences (O-LIFE; Mason, Claridge, & Jackson, 1995). These measures have played an important role in providing a comprehensive understanding of schizotypy expression, and offer several distinct benefits, such as being low-cost, brevity, non-intrusive, and easy to administer (Kwapil et al., 2018). The measures are effective in assessing schizotypy traits in both clinical and non-clinical samples (e.g., Kane et al., 2016; Rossi & Daneluzzo, 2002). Psychometrically examined schizotypy traits are associated with psychotic-like symptoms, paranoid symptoms, prodromal symptoms, and cognitive impairments (e.g., attentional deficits; Barrantes-Vidal et al., 2014; Kemp et al., 2021). Further, longitudinal studies have found that schizotypy dimensions can predict the development of schizophrenia spectrum disorders (e.g., Kwapil et al., 2013), highlighting the need to operationalise and examine schizotypy as a multidimensional construct. Alternatively, focusing only on one dimension can obscure *both* the nature of the associations with other schizotypy dimensions *and* potential insights into the aetiology and protective factors related to the development of schizophrenia and related disorders (Kwapil & Barrantes-Vidal, 2015). Therefore, it is important for studies to examine variables of interest (such as metacognitive beliefs in this paper) through the lens of multidimensional schizotypy.

#### Metacognitive Beliefs and Schizotypy

The relationships between metacognitive beliefs and schizotypy traits have been examined extensively in the literature. A common trend in the literature is to compare metacognitive beliefs in a non-clinical sample by artificially dichotomising/trichotomising them (e.g., high vs. low schizotypy or hallucination prone vs. non-hallucination prone using self-report measures). For instance, Chan et al. (2015) found that the high schizotypy group scored higher on all the MCQ-30 subscales than the low schizotypy group. Some studies have focussed on specific schizotypy traits (e.g., proneness to hallucinations and delusions) and have found that hallucination-prone participants scored higher on all the MCQ-65 subscales than non-hallucination-prone participants (e.g., Larøi & Van der Linden, 2005). Similarly, Larøi and Van der Linden (2005) found that delusion-prone participants scored higher on all the MCQ-65 subscales (except for cognitive confidence) than non-delusion-prone participants. Overall, prior empirical evidence suggests a greater endorsement of metacognitive beliefs in individuals with schizotypy traits.

Previous studies have examined the links of metacognitive beliefs with total schizotypy score and positive schizotypy, including its specific traits, such as proneness to delusions and hallucinations. For instance, the five metacognitive beliefs were found to be positively correlated with proneness to both hallucinations in general (Larøi et al., 2004) and auditory verbal hallucinations in particular (Jones & Fernyhough, 2006). Further, Larøi and Van der Linden (2005) found that negative beliefs about the uncontrollability and danger of thoughts, positive beliefs about worry, and negative beliefs about the need to control thoughts were significant predictors of delusion proneness. Similarly, Varese et al. (2011) found that all the metacognitive beliefs positively correlated with paranoid ideations. In regards to positive schizotypy, Debbané et al. (2009) found that all the metacognitive beliefs, except for cognitive self-consciousness, were significant predictors. In a similar vein, the five metacognitive beliefs were found to be positively correlated with the SPQ total score (Stirling et al., 2007). Overall, prior empirical evidence suggests the role of metacognitive beliefs in the expression of positive schizotypy (or its specific traits). It would be remiss not to mention that a study by Webster et al. (2022) was published around the final stages of our paper, found positive correlations of multidimensional schizotypy with three metacognitive beliefs (i.e., positive beliefs about worry, negative beliefs about the uncontrollability and danger of thoughts, and cognitive confidence). Although this study has limitations (see next section), it paved the way for viewing metacognitive beliefs through the lens of multidimensional schizotypy.

### Limitations of Prior Studies

Previous studies have paved the way for metacognitive beliefs–schizotypy research, but they have a few limitations. Firstly, the majority of these studies have relied on self-report measures without controlling for confounds associated with different responding styles, such as social desirability and randomisation/atypical responding, despite being recommended for controlling such biases (e.g., Nelson et al., 2013). Secondly, many studies have dichotomised/trichotomised samples using techniques like median splits. This kind of dichotomisation is often discouraged due to loss of effect size, power, and information about individual differences (Nelson et al., 2013). Thirdly, previous studies, except Webster et al. (2022) have mainly focused on the links of metacognitive beliefs with total schizotypy and positive schizotypy (or its specific traits, such as hallucination proneness). The failure to operationalise schizotypy as a multidimensional construct, including the computation of total schizotypy score, is usually not recommended as it results in a loss of information regarding links with various dimensions of schizotypy (Kwapil & Barrantes-Vidal, 2015).

Fourthly, studies have examined schizotypy traits using different self-report measures, such as the SPQ and the O-LIFE, which have several limitations. For instance, the SPQ examines a narrow expression of schizotypy (i.e., schizotypal personality disorder traits) and not the other manifestations of schizotypy, such as subclinical features and prodrome (Gross, Kwapil, Burgin, et al., 2018). This hinders the generalisability of the results for the schizotypy continuum. Further, the disorganisation dimension of the SPQ has been criticised for assessing disorganised features that are secondary to positive schizotypy rather than the actual disturbances in the ability to organise and express thoughts and behaviours (Gross et al., 2014). For instance, the SPQ item like, “People sometimes stare at me because of my odd appearance” (Raine, 1991, p. 557) could be endorsed due to volitional behaviours associated with positive schizotypy rather than cognitive-behavioural disorganisation (Gross et al., 2014). Similarly, the interpersonal dimension of the SPQ has been criticised for including items related to social anxiety and paranoia, which are not the key features of negative schizotypy (Gross et al., 2014). Constraining the focus on the O-LIFE, the Impulsive Non-Conformity subscale has been criticised for being a non-meaningful dimension of schizotypy as it tends to focus on impulsive, antisocial, and sensation-seeking traits, which are central to other personality disorders, such as borderline and antisocial personality disorders (Cochrane et al., 2010). Similarly, the Cognitive Disorganisation subscale of the O-LIFE has several items focusing on low self-esteem, neuroticism, or anxiety, which can be part of normal personality variation, and therefore, this subscale examines traits that are not specific to disorganised schizotypy (Cochrane et al., 2010; Kwapil et al., 2018). The Introvertive Anhedonia subscale of the O-LIFE has limited content coverage for negative schizotypy as it does not measure avolition, alogia, and flattened affect, which are key features of this dimension (Gross, Kwapil, Burgin, et al., 2018).

Finally, the study by Webster et al. (2022) did examine links between multidimensional schizotypy and metacognitive beliefs, but has several limitations, including (a) lack of control for social desirability biases, (b) focused only on three metacognitive beliefs (i.e., positive beliefs about worry, negative beliefs about the uncontrollability and danger of thoughts, and cognitive confidence), (c) no screening for psychiatric/neurological diagnosis, and (d) use of the O-LIFE (Short Version). Overall, previous studies suffered from empirical limitations related to the assessment of multidimensional schizotypy, which highlights the need for a newer measure that is in line with current conceptualisations of multidimensional schizotypy traits. Further, the links of metacognitive beliefs with negative schizotypy and disorganised schizotypy have not been examined in the existing literature extensively.

### The Current Study: Aim and Hypothesis

In the current study, we addressed the research gap related to the links of metacognitive beliefs with negative and disorganised schizotypy. Previous research has found metacognitive beliefs to be associated with cognitive difficulties in psychotic patients, and in those who are vulnerable to developing psychotic disorders (Brett et al., 2009). Similarly, in another study, Ostefjells et al. (2015) found cognitive self-consciousness to be associated with negative symptoms in psychotic patients. Thus, the empirical evidence indicates that metacognitive beliefs could be associated with other schizotypy dimensions, and not just with positive schizotypy. Further, we attempted to circumvent the limitations related to schizotypy measures employed in the previous studies by using the newly developed measure, the Multidimensional Schizotypy Scale (MSS; Kwapil et al., 2018). The MSS was developed to examine the current formulations of positive, negative, and disorganised schizotypy (Kwapil et al., 2018). The construction of the MSS involved a rigorous scale development methodology, including classical test theory, item response theory, and differential item functioning (Kwapil et al., 2018). The MSS has demonstrated good internal consistency, good test-retest reliability, good item discrimination, and minimal item bias for sex and ethnicity (Kwapil et al., 2018, 2021). Thus, the administration of the MSS is well-suited for our study as it assesses the current conceptualisation of multidimensional schizotypy, while also overcoming the conceptual and empirical limitations associated with schizotypy measures used in previous research. In the current study, the social desirability biases associated with self-report measures were examined and statistically controlled.

Overall, the current study aimed to assess the relationships of metacognitive beliefs with positive, negative, and disorganised schizotypy in a non-clinical sample. It was hypothesised that all five metacognitive beliefs would positively correlate with positive schizotypy. This hypothesis is based on previous studies, which found positive relationships between metacognitive beliefs and positive schizotypy, including its specific traits, such as proneness to hallucinations (e.g., Debbané et al., 2009; Varese et al., 2011). Considering there is limited evidence on the links between metacognitive beliefs and multidimensional schizotypy (Webster et al., 2022), there were no hypotheses formulated for the relationships of metacognitive beliefs with negative and disorganised schizotypy. Moreover, the study by Webster et al. (2022) was published around the final stages of our paper, after the data analysis was completed for our study, so we wanted to avoid HARKing (i.e., the practice of hypothesizing after results are known) in order to maintain the credibility of our research results (Murphy & Aguinis, 2019).

## Method

### Participants

This study was advertised to first-year psychology students and members of the wider community of Western Sydney University (e.g., post-graduate students). The recruitment for first-year psychology students was done through a web-based research participation system (i.e., SONA), whereas for community volunteers, the study was advertised at several physical locations of the university and on online platforms in the form of a flyer. The online screening session included a participant information sheet, consent form, and demographic information questionnaire. Once participants completed the screening session, they were invited to a laboratory session (or testing session) based on their responses on the demographic information questionnaire.

The inclusion criteria for this study were: (a) age (18–45 years), (b) being a native speaker of English or having at least 10 years of English education, and (c) no personal and family history (up to second degree) of psychological disorders, head trauma/injury and/or neurological illness (i.e., medical history). The age criterion was based on previous schizotypy studies, which mainly focus on younger subjects and/or those near the age range for higher risk of developing schizophrenia and related disorders (e.g., Kwapil et al., 2018). Further, this age criterion also assisted in avoiding age-related cognitive disruptions, which might impact the assessment of disorganised schizotypy (Kwapil et al., 2018). Subjects had no obligation to participate in the study, and therefore, were free to withdraw at any time without consequences. In the online session, 172 subjects participated, out of which 136 were eligible for the laboratory session. All participants were emailed the outcome of the online session along with the details of the laboratory session (if eligible). In the laboratory session, 76 subjects participated and received either course credits (for first-year psychology students), or a $40 AUD Coles Group & Myer gift card (for the community volunteers) as compensation for their time. This study was approved by Western Sydney University Human Research Ethics Committee and Bond University Human Research Ethics Committee (Ethics Application Number: H12881).

### Measures

#### Demographic Information Questionnaire

This questionnaire elicited information related to age, gender, English language proficiency, highest level of completed education, ethnic background, and personal and family history of psychological disorders, head trauma/injury and/or neurological illness.

#### Multidimensional Schizotypy Scale (MSS)

The MSS is a self-report measure consisting of 77 true-false items, which are divided into three subscales: positive (26 items), negative (26 items), and disorganised (25 items) schizotypy, respectively (Kwapil et al., 2018, 2021). Each of the subscales have good to excellent internal consistency reliability; α was .89 for positive and negative schizotypy and α was .94 for disorganised schizotypy (Kwapil et al., 2018, 2021). Further, the MSS demonstrated a strong test-retest reliability based on intraclass correlation values, .84 for positive schizotypy, .90 for negative schizotypy, and .85 for disorganised schizotypy (Kwapil et al., 2021). The scoring of the MSS involves summing the number of items endorsed in the schizotypic direction on each subscale (Kwapil et al., 2021). The range of raw scores for positive and negative schizotypy subscales is 0 to 26, and for disorganised schizotypy subscale is 0 to 25. There is also considerable empirical evidence supporting the construct validity of the MSS, including studies using interviews, questionnaires, and experience sampling methodology/ambulatory assessment (Kwapil et al., 2021). In line with the fully dimensional view of schizotypy and recommendations of Gross, Kwapil, Raulin, et al. (2018) and Kwapil et al. (2018), we used the MSS subscales’ scores as continuous variables as opposed to using arbitrary cut-off scores or a total schizotypy score.

#### Marlowe-Crowne Social Desirability Scale (MCSDS)

The MCSDS is a 33-true-false-item scale for measuring social desirability (Crowne & Marlowe, 1960). This scale has good internal consistency (.88) and test-retest reliability correlation (.89), respectively (Crowne & Marlowe, 1960). The possible range of scores on this scale is from 0 to 33, with a higher score indicating a tendency towards a more socially desirable response. This scale was used to control the effect of social desirability on self-report measures of schizotypy and metacognitive beliefs, as controlling for such biases is recommended in the literature, especially in schizotypy studies (DeVylder & Hilimire, 2015).

#### Metacognitions Questionnaire-30 (MCQ-30)

The MCQ-30 is a 30-item self-report for measuring metacognitive beliefs (Wells & Cartwright-Hatton, 2004). Each item on the MCQ-30 is rated on a 4-point Likert scale, ranging from 1 (*do not agree*) to 4 (*agree very much*). The MCQ-30 has five separate subscales: (a) Positive Beliefs about Worry, (b) Negative Beliefs about the Uncontrollability and Danger of Thoughts, (c) Cognitive Confidence, (d) Negative Beliefs about the Need to Control Thoughts, and (e) Cognitive Self-Consciousness (Wells & Cartwright-Hatton, 2004). Each subscale consists of 6 items. Higher scores on each subscale indicate a greater endorsement of maladaptive metacognitive beliefs. The scores on each subscale are added to calculate the total score. The range of raw scores for each subscale is 6 to 24, and for the total score is 30 to 120. The five subscales have good to excellent internal consistency reliability; α ranges from .72 to .93 (Wells & Cartwright-Hatton, 2004). The MCQ-30 has also demonstrated good convergent validity and acceptable to good test-retest reliability (Wells & Cartwright-Hatton, 2004).

### Procedure

This study consisted of two sessions: An online screening session and a laboratory session (or testing session). Participants were screened after the online session, and eligible ones were invited to the testing session. The online screening and testing sessions lasted approximately 10 minutes and 90 minutes, respectively. Considering the longer duration of the testing session, participants were allowed to take a break whenever they felt tired. In the testing session, participants were tested individually in a quiet environment, and the researcher (AG or MR) was always present in the room. To ensure transparency in reporting procedural details, the sequence of all the administered measures is provided in this section. While the Short Stress State Questionnaire (SSSQ; pre and post forms) and the Contrast Discrimination Task (CDT) were administered, the data from these measures is not reported in this study as it pertains to a different project. Participants first completed the SSSQ (pre-form), which was followed by the MSS and the MCSDS. The personality assessment was followed by the administration of the metacognition measures in a counterbalanced order: the MCQ-30 and the CDT, followed by the SSSQ (post-form). All the self-report measures in the testing session were presented on Qualtrics. After completing the testing session, participants were debriefed about the assessment of schizotypy and were given credit points/gift cards immediately.

## Statistical Analyses

In this study, data were analysed using IBM SPSS Statistics (Version 25). In the final analyses, data from 68 participants were included. Eight participants were removed from the analyses as five attempted to expedite the testing session by not reading instructions of the measures and/or by engaging in random responding. After completing the testing session, one participant reported about their recent diagnosis of a psychological disorder, and for two participants, there was a technical error during the completion of the testing session.

Prior to conducting primary analyses, the data were extensively screened for outliers and normality. There were no significant univariate outliers in the data; that is, *z*-scores were < ±3.29, based on the guidelines given by Tabachnick and Fidell (2013). The normality assumption was examined using skewness and kurtosis values for medium-sized samples (50 ≤ *N* ≤ 300); (Kim, 2013; Tabachnick & Fidell, 2013). Therefore, *z*-scores (*z*_*skewness*_ and *z*_*kurtosis*_) were calculated, and data was considered non-normally distributed when the *z*-scores values for skewness and/or kurtosis were greater than 3.29 (Kim, 2013). Thus, using this criterion, the normality assumption was met for most of the variables, except for the two MSS schizotypy subscales (i.e., negative and disorganised schizotypy).

For descriptive purposes, the mean estimates and standard deviations were calculated for the self-report measures and age. Spearman’s rank correlations were conducted to assess the relationships of the MSS subscales with the MCQ-30 subscales. This correlational technique was also used to assess the relationships of social desirability with the MSS subscales and the MCQ-30 subscales, respectively, to determine if social desirability was a covariate. The Spearman rank-order correlation was used because its superior performance in terms of variability and robustness for non-normally distributed data (de Winter et al., 2016). Spearman’s partial correlations were also conducted to assess the relationships of the MSS subscales with the MCQ-30 subscales, while controlling for social desirability. Similarly, Spearman’s semi-partial correlations (or part correlations) were conducted to assess correlation between two variables of interest, while controlling for social desirability only from one variable. All tests in this study were conducted using two-tailed criteria (*p* < .05).

## Results

### Sample Characteristics

Table 1 presents descriptive statistics for all the self-report measures used in this study, and Table 2 presents the demographic characteristics of the sample.

**Table 1.** Descriptive Statistics of Self-Report Measures (*N* = 68).

Measures	*M* (*SD*)
Multidimensional Schizotypy Scale	
Positive Schizotypy	4.71 (3.89)
Negative Schizotypy	4.09 (3.78)
Disorganised Schizotypy	3.90 (4.98)
Marlowe-Crowne Social Desirability Scale	17.49 (5.66)
Metacognitions Questionnaire-30	
PBW	12.21 (4.76)
NBUD	12.53 (4.12)
CC	11.18 (3.64)
NTC	12.88 (3.83)
CSC	16.71 (4.15)
Total score	65.50 (12.04)

*Note.* PBW = Positive Beliefs about Worry; NBUD = Negative Beliefs about the Uncontrollability and Danger of Thoughts; CC = Cognitive Confidence; NTC = Negative Beliefs about the Need to Control Thoughts; CSC = Cognitive Self-Consciousness.

**Table 2.** Demographic Characteristics of the Sample (*N* = 68).

Characteristic	Full sample
Age (in years)^a^	24.09 (5.4)
Gender^b^	
Male	26 (38.2%)
Female	42 (61.8%)
Educational qualification (completed)^b^	
Completed high school	24 (35.3%)
College/University	44 (64.7%)
English proficiency^b^	
Native speakers	44 (64.7%)
Non-native speakers^c^	24 (35.3%)
Ethnicity^b^	
Oceanian	6 (8.8%)
European	15 (22.1%)
African	1 (1.5%)
Middle Eastern	7 (10.3%)
East Asian	9 (13.2%)
South Asian	16 (23.5%)
People of the Americas	4 (5.9%)
Mixed	10 (14.7%)

^a^Mean age is reported with standard deviation in parenthesis.

^b^Demographic data are reported as the number of cases with the percentage in parentheses.

^c^The participants had a minimum of 10 years of English education (self-reported).

### Correlations Among Multidimensional Schizotypy, Metacognitive Beliefs, and Social Desirability

Table 3 presents correlations among multidimensional schizotypy, social desirability, and metacognitive beliefs, including the effect sizes based on the guidelines given by Cohen (1988). Among these variables, social desirability negatively correlated with disorganised schizotypy (medium effect) and metacognitive beliefs, including negative beliefs about the uncontrollability and danger of thoughts, cognitive confidence, negative beliefs about the need to control thoughts, and the MCQ-30 total score (small to medium effects). Therefore, Spearman’s partial/semi-partial correlations were conducted (see also Table 3).

**Table 3.** Correlations Among Multidimensional Schizotypy, Social Desirability, and Metacognitive Beliefs.

Variable	SDE	Metacognitive beliefs					
		PBW	NBUD	CC	NTC	CSC	MCQ-30 total score
PS	−.18	.36**	.45*** (.41***)^a^	.24* (.18)^a^	.41*** (.36**)^a^	.01	.49*** (.44***)^a^
NS	−.09	.11	.28* (.29*)^a^	.34** (.33**)^a^	.26* (.24*)^a^	−.03	.30* (.30*)^a^
DS	−.33**	.15(.09)^b^	**.** ***62*** *** (. ***56*** ***)^c^	.24* (.17)^c^	.27* (.15)^c^	−.17 (−.15)^b^	.37** (.25*)^c^
SDE	—	−.12	−.38**	−.29*	−.34**	.12	−.37**

*Note.* Zero-order Spearman’s rank correlation coefficients are presented without the parentheses, and Spearman’s rank partial/part correlation coefficients are presented in the parentheses. Medium effect sizes are presented in bold, and large effect size is presented in bold and italics. SDE = social desirability; PBW = Positive Beliefs about Worry; NBUD = Negative Beliefs about the Uncontrollability and Danger of Thoughts; CC = Cognitive Confidence; NTC = Negative Beliefs about the Need to ControlThoughts; CSC = Cognitive Self-Consciousness; MCQ-30 = Metacognitions Questionnaire-30; PS = Positive Schizotypy; NS = Negative Schizotypy; DS = Disorganised Schizotypy

^a^Part correlations controlling for social desirability from the MCQ-30 subscales, including the MCQ-30 total score.

^b^Part correlations controlling for social desirability from disorganised schizotypy.

^c^Partial correlations controlling for social desirability from both disorganised schizotypy and the MCQ-30 subscales, including the MCQ-30 total score.

*p < .05. **p < .01. ***p < .001.

Positive schizotypy was positively correlated with all the metacognitive beliefs, except for cognitive self-consciousness (small to medium effects). The correlations with positive schizotypy remained significant after controlling for social desirability from negative beliefs about the uncontrollability and danger of thoughts, negative beliefs about the need to control thoughts, and the MCQ-30 total score (medium effects). However, the correlation between positive schizotypy and cognitive confidence was no longer significant after controlling for social desirability from cognitive confidence.

Negative schizotypy was positively correlated with all the metacognitive beliefs, except for positive beliefs about worry and cognitive self-consciousness (small to medium effects). All the correlations with negative schizotypy remained significant after controlling for social desirability from negative beliefs about the uncontrollability and danger of thoughts, cognitive confidence, negative beliefs about the need to control thoughts, and the MCQ-30 total score (medium effects).

Disorganised schizotypy was positively correlated with all the metacognitive beliefs, except for positive beliefs about worry and cognitive self-consciousness (small to large effects). After controlling for social desirability from disorganised schizotypy and all the metacognitive beliefs (except for positive beliefs about worry and cognitive self-consciousness), the results were as follows: (a) positive correlations of disorganised schizotypy remained significant with negative beliefs about the uncontrollability and danger of thoughts (large effect) and the MCQ-30 total score (small effect), and (b) positive correlations of disorganised schizotypy with cognitive confidence and negative beliefs about the need to control thoughts were no longer significant. Overall, metacognitive beliefs positively correlated with multidimensional schizotypy.

## Discussion

In this study, we examined the relationships between multidimensional schizotypy and metacognitive beliefs in a non-clinical sample. Previous studies have mainly examined the relationships of metacognitive beliefs with total schizotypy score and/or positive schizotypy only (including its specific traits). The current study extends the literature by examining the relationships of metacognitive beliefs with disorganised and negative schizotypy. After controlling for social desirability, the results were as follows: (a) positive schizotypy was positively correlated with all the metacognitive beliefs, except for cognitive self-consciousness and cognitive confidence, (b) negative schizotypy was positively correlated with all the metacognitive beliefs, except for positive beliefs about worry and cognitive self-consciousness, (c) disorganised schizotypy was positively correlated with negative beliefs about the uncontrollability and danger of thoughts, but not other metacognitive beliefs, and (d) the MCQ-30 total score was positively correlated with positive, negative, and disorganised schizotypy. Below, the findings of the current study are explained in the context of prior literature, followed by limitations, and conclusions, including suggestions for future research on metacognitive beliefs and multidimensional schizotypy.

### The Relationships Between Metacognitive Beliefs and Multidimensional Schizotypy

Results revealed positive correlations between the different types of metacognitive beliefs and multidimensional schizotypy. A positive correlation was also observed between the MCQ-30 total score and multidimensional schizotypy, which suggests that endorsement of metacognitive beliefs is related to positive, negative, and disorganised schizotypy expression. In regards to positive schizotypy, after controlling for social desirability, results revealed positive correlations with positive beliefs about worry, negative beliefs about the uncontrollability and danger of thoughts, and negative beliefs about the need to control thoughts, respectively. These results are in line with previous studies, which examined the links of metacognitive beliefs with positive schizotypy (e.g., Debbané et al., 2009; Webster et al., 2022). Overall, the observed links between metacognitive beliefs and positive schizotypy partially supported our hypothesis that all five metacognitive beliefs would positively correlate with positive schizotypy.

A zero-order correlation revealed a positive relationship between cognitive confidence and positive schizotypy. However, this correlation was no longer significant after controlling for social desirability from cognitive confidence. This null finding suggests that cognitive confidence is not related to positive schizotypy, at least not under the circumstances tested in the current study. We also observed no correlation between cognitive self-consciousness and positive schizotypy, which is consistent with a previous study by Debbané et al. (2009).

By focusing on negative schizotypy, positive correlations were observed with negative beliefs about the uncontrollability and danger of thoughts, cognitive confidence, and negative beliefs about the need to control thoughts, respectively. These findings remained significant, even after controlling for social desirability from the metacognitive beliefs. These findings are novel, as previous studies have mainly focused on positive schizotypy and/or total schizotypy score. A study by Webster et al. (2022) found positive correlations of negative schizotypy with three metacognitive beliefs (i.e., positive beliefs about worry, negative beliefs about the uncontrollability and danger of thoughts, and cognitive confidence). Thus, our findings are somewhat consistent with Webster et al. (2022), except for the links with the positive beliefs about worry and the negative beliefs about the need to control thoughts. Specifically, we observed a positive correlation between negative schizotypy and negative beliefs about the need to control thoughts, which is a novel finding as Webster et al. (2022) did not focus on this metacognitive belief. On the contrary, we observed no correlations of negative schizotypy with positive beliefs about worry and cognitive self-consciousness, which suggests no links of negative schizotypy with these metacognitive beliefs. Overall, our results extend the metacognitive beliefs–schizotypy literature by examining and observing the relationships between metacognitive beliefs and negative schizotypy.

For disorganised schizotypy, positive correlations were observed with all the metacognitive beliefs, except for the positive beliefs about worry and cognitive self-consciousness. The null results suggest that disorganised schizotypy is not linked with these metacognitive beliefs, at least not under the circumstances tested in the current study. Notably, among the schizotypy dimensions, only disorganised schizotypy was found to be negatively correlated with social desirability. This finding is somewhat consistent with the validation study for the MSS, in which social desirability was found to be negatively correlated with positive, negative, and disorganised schizotypy (Kwapil et al., 2018). Our results suggest that individuals who give more socially desirable responses are less likely to endorse negative schizotypy items. Therefore, we statistically controlled for the influence of social desirability on disorganised schizotypy to determine the precise links with metacognitive beliefs.

After controlling for social desirability, disorganised schizotypy was found to be correlated only with negative beliefs about the uncontrollability and danger of thoughts, which is consistent with the finding of Webster et al. (2022). We also observed that after controlling for social desirability, disorganised schizotypy was no longer correlated with cognitive confidence and negative beliefs about the need to control thoughts, indicating no links of disorganised schizotypy with these metacognitive beliefs.

The correlation of negative beliefs about the uncontrollability and danger of thoughts was strongest with disorganised schizotypy (large effect) as compared to positive schizotypy and negative schizotypy (small to medium effects). This finding reflects that disturbances in the organisation and expression of thoughts, speech, and behaviour might make one strongly believe that their thoughts are indeed uncontrollable and dangerous in comparison to when someone who experiences distorted functioning of normal processes (positive schizotypy) or deficits in one’s normal behaviour (negative schizotypy). Overall, our findings highlight the need to operationalise and examine schizotypy as a multidimensional construct (Kwapil & Barrantes-Vidal, 2015). Otherwise, focusing only on positive schizotypy obscures the insights into the links of metacognitive beliefs with disorganised and negative schizotypy.

Our study has implications for future research and practice. In line with the S-REF model, which posits the role of maladaptive metacognitive beliefs in predisposition to psychopathology (Sellers et al., 2016), our findings suggest that the metacognitive beliefs might play a role in the exacerbation of positive, negative, and disorganised schizotypy expression, respectively. Alternatively, a greater endorsement of metacognitive beliefs could be the direct manifestation of multidimensional schizotypy expression. Future studies could examine these causal possibilities, and this line of research can be informed by previous studies which examined the effects of experimental manipulation of metacognitive beliefs on paranoia (Sellers et al., 2018). From a treatment standpoint, our study has implications for research on the modification of maladaptive metacognitive beliefs for the prevention of mental health difficulties, such as metacognitive training. Instead of focusing only on traits of positive schizotypy, our results suggests that researchers could focus on negative and disorganised schizotypy traits when examining efficacy of the metacognitive training for prevention of schizophrenia/psychosis. Future studies could formulate the S-REF Model in the context of multidimensional schizotypy, as the model currently focuses on clinical conditions or general psychopathology. Overall, our study extends the metacognitive beliefs literature in the context of multidimensional schizotypy.

### Limitations and Future Directions

Our study has some limitations. First, the current study used a correlational research design, which does not provide insights into causal links between multidimensional schizotypy and metacognitive beliefs. However, the results of the current study provide a promising entry point for examining the causal possibilities discussed earlier. Second, considering the scarcity of research on links of metacognitive beliefs with negative and disorganised schizotypy, our findings are preliminary and are based on a medium sample size. Despite this, we were able to observe medium to large effect sizes for most of the correlational findings in the current study. Nevertheless, we recommend replicating this study in a larger sample to confirm or refute our findings. Third, the participant’s mental health was not formally assessed, which could have influenced the profiles of multidimensional schizotypy and metacognitive beliefs, including their relationships in the current study. For instance, previous studies have found higher scores on multidimensional schizotypy in clinical samples (e.g., bipolar disorders and depression) as compared to healthy controls (e.g., Rossi & Daneluzzo, 2002). It is worth noting that most of the previous studies on schizotypy and/or metacognitive beliefs did not focus on examining the mental health of the participants either (e.g., Chan et al., 2015). Therefore, future studies could investigate whether mental health status influences the metacognitive beliefs and multidimensional schizotypy dynamics. Fourth, we note that majority of the participants were university students, which limits the generalisability of the findings. This limitation can be stated for most of the schizotypy studies, as the recruitment of university students is a common practice in the literature. In comparison to a community sample, university students are considered healthy and cognitively intact enough to study at a tertiary level (Kane et al., 2016). Thus, inclusion of university students in our study could have contributed to the conservative links between metacognitive beliefs and multidimensional schizotypy. At the same time, the recruitment of university students offers several advantages, such as they are at or near the age of greatest risk for displaying schizophrenia spectrum symptomatology (Kwapil et al., 2018). Future studies can therefore examine whether the results of the current study can be generalised to a more representative community sample. Another direction for future research could be examining the links between metacognitive beliefs and multidimensional schizotypy using the experience sampling method. This method seems to be an attractive option for researchers as it offers several advantages (e.g., less reliance on retrospective recall), and could assist in understanding the interplay between the metacognitive beliefs and schizotypy expression in daily life.

## Conclusions

In this study, metacognitive beliefs were positively correlated with positive, negative, and disorganised schizotypy in a non-clinical sample. This study was the first attempt in the literature to examine these links, unlike previous studies focusing *only* on the links of metacognitive beliefs with positive schizotypy and/or total schizotypy score. We attempted to circumvent the limitations related to schizotypy assessment, evident in previous studies, by using a newly developed measure, the MSS, which was based on the current operationalisations of schizotypy dimensions given by Kwapil et al. (2018). Overall, our study extends the literature by examining the relationships of metacognitive beliefs with negative and disorganised schizotypy, and thus, has implications for metacognition research and treatment modalities.

## Data Availability

Data supporting the results are available by contacting the corresponding author.*
